# Nuclear translocation of telomerase reverse transcriptase is a critical process in lymphatic metastasis of nasopharyngeal carcinoma

**DOI:** 10.3892/ol.2014.2689

**Published:** 2014-11-07

**Authors:** TING-TING WU, CHEN CHEN, SHI-MING CHEN, YONG XU, YAN WANG, ZHE CHEN, FEI WANG, BO-KUI XIAO, ZE-ZHANG TAO

**Affiliations:** 1 Department of Otolaryngology-Head and Neck Surgery, Zhongnan Hospital of Wuhan University, Wuhan, Hubei 430071, P.R. China; 2 Department of Otolaryngology-Head and Neck Surgery, Renmin Hospital of Wuhan University, Wuhan, Hubei 430060, P.R. China

**Keywords:** nasopharyngeal carcinoma, metastasis, translocation, telomerase reverse transcriptase

## Abstract

Telomerase reverse transcriptase (TERT) is the predominant functional unit of telomerase and maintains the telomere length and the stability of chromosomes. Recently, TERT has been shown to be a critical factor in a number of other biological processes, including cell proliferation and cancer metastasis. In addition, although numerous studies have been conducted, the subcellular localization of the TERT protein and the association of such with cancer metastasis remains unclear. To investigate the involvement of TERT in *in vivo* metastasis, quantum dots-based immunofluorescence and western blot analysis were conducted to detect changes in the subcellular localization of TERT in human nasopharyngeal carcinoma (NPC) tissues and metastatic lymph nodes. To further investigate, metastatic and non-metastatic models of NPC were generated using 5–8F (high metastasis capability) and 6–10B (low metastasis capability) cell lines, respectively. It was found that TERT protein was overexpressed in NPC tissue samples and metastatic lymph nodes and TERT was predominantly located in the cytoplasm of primary NPC tissues, while TERT was predominantly located in the nucleus of the metastatic lymph nodes. The ratio of cytoplasmic TERT/nuclear TERT for the primary tumor of the 6–10B cell line was almost six-fold higher than that of the metastatic lymph nodes of the 5–8F cell line. TERT translocation from the cytoplasm to nucleus may present a critical step in the lymphatic metastasis of NPC. Thus, TERT translocation may be more useful than TERT expression level and telomerase activity for predicting the metastasis of NPC.

## Introduction

Telomerase is a ribonucleoprotein complex, the predominant function of which is to add six nucleotide repeats (TTAGGG) to the end of chromosomes, in a mechanism that is dependent on telomerase reverse transcriptase (TERT) and intrinsic RNA template (TERC) activity, as well as additional associated proteins. This process compensates for the telomere loss that accompanies cell division and chromosome replication, and thus prolongs the telomere length-restricted replicative lifespan of cells (1,2). In contrast to the majority of normal human somatic cells, which do not express telomerase and eventually enter into senescence when telomeres shorten to a crucial point, >80% of human cancers exhibit a high level of telomerase activity, which maintains telomere length. TERC is ubiquitously expressed in all normal and cancer cells, whereas TERT, which is involved in cellular immortalization and carcinogenesis, acts as a rate-limiting factor for the activation of telomerase (3,4). Recently, several additional activities exhibited by TERT have been identified, which indicates that TERT may exhibit telomere-independent biological functions, including the promotion of cell proliferation (5,6), extension of cell life (6,7), delaying cell aging (8,9) and modulation of cell differentiation (8). A number of these novel functions do not rely on the reverse transcriptase activity of TERT (7,10).

TERT protein expression is regulated by a complicated system, predominantly involving transcriptional and translational control (11–13). TERT translational control has been demonstrated to be critical for functional regulation due to its subcellular location (14). The dynamic subcellular location of TERT is dependent on the cell cycle, DNA damage or cellular transformation (15). Notably, a number of studies have shown that TERT, which is regarded as a nuclear protein, has been identified not only in the nucleus but also occasionally in the cytoplasm (14,16,17). However, at present, the biological significance of the TERT subcellular location in the process of *in vivo* lymphatic metastasis of nasopharyngeal carcinoma (NPC) remains unclear.

## Materials and methods

### Cell lines and reagents

The human NPC cell lines, 5–8F (high metastasis capability) and 6–10B (low metastasis capability), were purchased from China Center for Type Culture Collection (Wuhan, China) and conserved at Renmin Hospital of Wuhan University (Wuhan, China) and stored in liquid nitrogen. Fetal bovine serum was obtained from Thermo Fisher Scientific (Waltham, MA, USA). RPMI 1640 medium and 0.25% trypsin solution were purchased from Invitrogen Life Technologies (Carlsbad, CA, USA). The TERT antibody (AB5181) was purchased from Abcam (Cambridge, UK) and the glyceraldehyde 3-phosphate dehydrogenase (GAPDH) antibody (5174) was purchased from Cell Signaling Technology, Inc., (Beverly, MA, USA). The Quantum dots (QDS) immunofluorescence detection kit was purchased from Wuhan Jiayuan Quantum Dots Co., Ltd., (Wuhan, China).

### Cell culture

The 5–8F and 6–10B cell lines were cultured in RMPI 1640 medium supplemented with 10% fetal bovine serum, 10 μg/ml ampicillin and 10 μg/ml kanamycin, and incubated at 37°C in a humidified atmosphere of 5% CO_2_.

### Human NPC tissue samples

A total of 39 human NPC tissue samples and 13 lymph nodes were obtained from NPC cancer patients undergoing biopsy at Renmin Hospital of Wuhan University and the diagnosis of NPC was confirmed by pathological examination. Paraffin blocks created from these biopsies were used to construct tissue microarrays. Written informed consent was obtained from all patients.

### Xenograft model

Female BALB/c nude mice (four to six weeks old) were obtained from Beijing HFK Bioscience Co., Ltd., (Beijing, China) and quarantined for one week prior to tumor implantation. The xenograft tumor model was established by subcutaneously injecting 5–8F and 6–10B cells (2×10^6^) suspended in 0.2 ml RPMI 1640 medium into the right flank of the mice. Twelve weeks following implantation, the mice were sacrificed and the primary tumors and the draining lymph nodes were collected for western blot analysis. Animal welfare and experimental procedures were followed strictly. This study was approved by the ethics committee of Renmin Hospital of Wuhan University.

### Western blot analysis

Total cell lysate was performed according to standard instructions. Cytosol and nuclear extracts were prepared following the manufacturer’s instructions. The lysates were resolved using 10% SDS-PAGE, transferred to nitrocellulose membranes and immunoblotted with primary antibodies against TERT and GAPDH. Following incubation with secondary antibodies, the protein bands were detected using an enhanced chemiluminescence reagent (Thermo Fisher Scientific, Rockford, IL, USA).

### QDs based immunofluorescence

TERT immunofluorescence staining using a 545-QD-SA probe (Wuhan Jiayuan Quantum Dot Technological Development Co., Ltd., Wuhan, China) was performed on the NPC tissue and metastatic lymph nodes. The slide was deparaffinized, antigen retrieval was performed, blocked with 3% bovine serum albumin and incubated with primary mouse anti-human TERT monoclonal antibody. The slide was then washed and incubated with biotinylated goat anti-mouse IgG, washed, blocked and incubated with 545-QD-SA, mounted and observed by fluorescence microscopy. Images were captured and analyzed by Nuance 2.10 software (CRi, Woburn, MA, USA) (18).

### Telomerase repeat amplification protocol (TRAP) assay of telomerase activity

TRAP assays were performed using the Telo TAGGG Telomerase polymerase chain reaction ELISA kit (Roche, Mannheim, Germany) according to the manufacturer’s instructions. The relative telomerase activity was calculated using the following formula: Relative telomerase activity (%) = sample A450 nm-A690 nm unit/positive control A450 nm-A690 nm unit. The mean value was calculated from three independent experiments.

### Statistical analysis

All data are expressed as the mean ± standard deviation. One-way analysis of variance was performed using SPSS version 13.0 (SPSS Inc., Chicago, IL, USA) and P<0.05 was considered to indicate a statistically significant difference.

## Results

### Nuclear translocation of TERT is associated with the lymphatic metastasis of NPC

**Figure 1:**
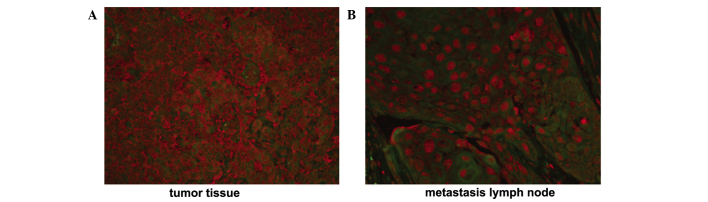
TERT protein expression levels were analyzed by quantum dots-based immunofluorescence (magnification, ×400). (A) TERT protein expression was identified in nasopharyngeal carcinoma primary tumor (red), whereby TERT was positive in 34/39 tested samples and was predominantly located in the cytoplasm. (B) TERT protein expression was identified in the metastatic lymph nodes of nasopharyngeal carcinoma (red), whereby TERT was positive in 13/13 tested samples and the signal of TERT was predominantly located in the nucleus. TERT, telomerase reverse transcriptase.

To investigate the subcellular localization of the TERT protein and determine whether lymphatic metastasis of NPC is accompanied by changes in TERT localization, the TERT protein expression was analyzed by QDS-based immunofluorescence and western blot analysis, respectively. A positive TERT staining signal was detected in 34/39 NPC tissue samples and 13/13 metastatic lymph nodes, identified in the cytoplasm and nucleus. In NPC tissue samples, TERT protein was exclusively localized to the cytoplasm, with a weak positive signal identified in the nucleus. (Fig. 1A) By contrast, the TERT protein was translocated to the nucleus from the cytoplasm when NPC cells metastasized to lymph nodes (Fig. 1B).

**Figure 2:**
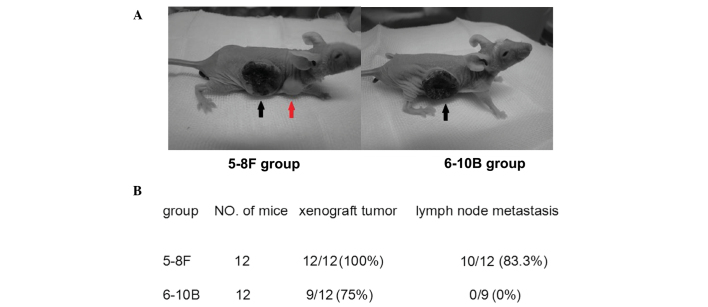
Xenograft tumor models of nasopharyngeal carcinoma implanted with the 5–8F and 6–10B cell lines. (A) Images captured of the mice used for the xenograft tumor model. For the 5–8F group, the black arrow indicates the primary tumor and the red arrow shows the metastatic lymph node of the primary tumor. For the 6–10B group, the black arrow shows the primary tumor and no metastasis of the lymph node was identified. (B) The table shows the rate of tumor formation and metastasis of the two groups.

**Figure 3:**
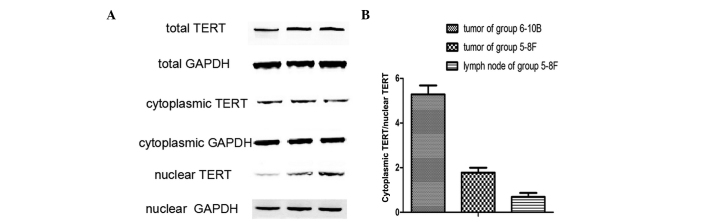
Western blot analysis. (A) Total, cytoplasmic and nuclear TERT protein expression levels were identified by western blot analysis. Total extract, cytoplasmic extract and nuclear extract of the primary tumor for the 5–8F and 6–10B groups, as well as the metastatic lymph nodes of group 5–8F were analyzed for the TERT expression level using an anti-TERT antibody and GAPDH was used as the internal control. (B) The ratio of cytoplasmic TERT/nuclear TERT in the primary tumors of the 5–8F and 6–10B groups, as well as the metastatic lymph node of group 5–8F. TERT, telomerase reverse transcriptase; GAPDH, glyceraldehyde 3-phosphate dehydrogenase.

To further confirm the subcellular localization of TERT in NPC metastasis, TERT protein expression was analyzed in non-metastatic xenograft tumor tissues, metastatic tumor tissues and metastatic lymph nodes by western blot analysis. The TERT protein was detected in all samples (Fig. 2), and the ratio of cytoplasmic TERT/nuclear TERT differed between the three groups. (Fig. 3A) A significant difference was identified between the ratios of cytoplasmic TERT/nuclear TERT in non-metastatic xenograft tumor tissues, metastatic tumor tissues and metastatic lymph nodes (Fig. 3B). Thus, nuclear translocation of TERT was closely associated with the lymphatic metastasis of NPC.

### Increased telomerase activity in the translocation of TERT

**Figure 4:**
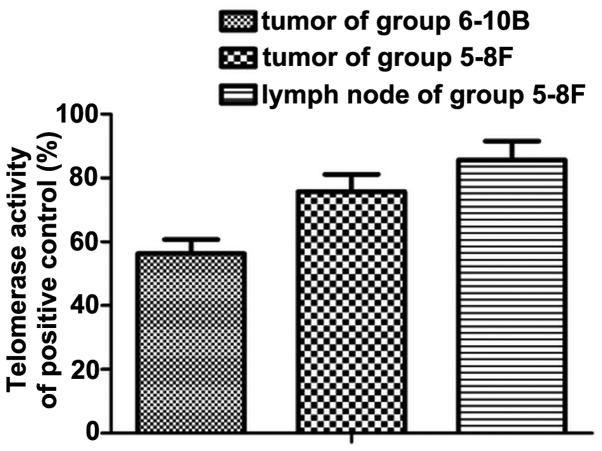
Telomerase activity in the primary tumor and metastatic lymph nodes. TA was measured by telomerase repeat amplification protocol assay and TA was low in the primary tumor of group 6–10B, which did not metastasize. TA was moderate in the primary tumor of group 5–8F and was high in the metastatic lymph nodes of group 5–8F. TA, telomerase activity.

Telomerase activity in all three groups was detected using TRAP-ELISA. Consistent with the levels of nuclear TERT protein identified, telomerase activity was low in the non-metastatic primary tumor. In addition to the translocation of the TERT protein, high telomerase activity was maintained and reached a level, which was significantly different to that detected in metastatic lymph nodes (P<0.05; Fig. 4).

## Discussion

In this study, the TERT protein expression level, subcellular localization and telomerase activity in the process of NPC lymphatic metastasis were investigated. It was demonstrated that TERT protein expression level was increased in NPC tumor tissue and metastatic lymph nodes. Furthermore, the TERT subcellular location was associated with telomerase activity, and nuclear translocation of TERT was associated with lymphatic metastasis of NPC.

As hypothesized, increased TERT protein expression was observed in NPC tumor tissue and metastatic lymph nodes. Furthermore, nuclear translocation of TERT may be involved in the regulation of telomerase activity and the lymphatic metastasis of NPC.

As telomerase activity is controlled by TERT, and the association between NPC metastasis and TERT expression levels and TERT subcellular localization remains unclear (14), the cellular localization of TERT in the process of NPC metastasis was investigated. In the present study, TERT protein expression levels and telomerase activity were increased significantly in NPC tissues and metastatic lymph nodes and lymphatic metastasis was observed to be closely associated with the nuclear translocation of TERT, which was detected by QDS-based immunofluorescence and western blot analysis.

In the xenograft tumor model of NPC, TERT protein expression levels and telomerase activity were analyzed in all xenograft tumor tissue and metastasis lymph nodes, TERT was predominantly distributed in the cytoplasm in xenograft tumor tissues of the non-metastatic group, which is important for protecting cells from apoptosis stimuli (19–21). TERT was predominantly distributed in the nucleus in metastatic lymph nodes. These results indicated that nuclear translocation of TERT increases the telomerase activity and lymphatic metastasis of NPC cells. In metastatic lymph nodes, nuclear translocation of TERT may be recharacterized to promote invasion and metastasis of NPC cells. It has been proposed that TERT may be involved in altering gene expression of proteins associated with invasion and metastasis of NPC, including TGF-β and β-catenin (22).

In normal cells, only phosphorylated TERT regulates telomerase activity following nuclear translocation, however, tumor cells that constitutively exhibit high levels of telomerase activity express the TERT protein in the phosphorylated form, which is located in the nucleus (16). Recent studies have shown that following phosphorylation by Akt and protein kinase C, TERT was exported to the nucleus, playing its role in maintaining telomerase activity (23). No methods were available to directly detect phosphorylated nuclear TERT in tissue samples, therefore, additional studies are required to confirm the expression of nuclear TERT identified in the study. However, the potential involvement of phosphorylation during the process of lymphatic metastasis of NPC must not be ignored.

In conclusion, TERT protein expression and telomerase activity are increased in NPC tissues. In comparison with the TERT protein expression levels, the nuclear translocation of TERT may be more important in the regulation of telomerase activity and lymphatic metastasis of NPC. Therefore, TERT nuclear translocation alone or in combination with telomerase activity or TERT expression level may present an appropriate biomarker for predicting the lymphatic metastasis of NPC.
